# Barriers to interval cholecystectomy following percutaneous cholecystostomy in patients with acute calculous cholecystitis

**DOI:** 10.1007/s00464-025-12161-x

**Published:** 2025-09-22

**Authors:** Sourav Podder, Kirsten Lung, George Ibrahim, Scott Koeneman, Joshua Marks, Murray Cohen, Anirudh Kohli

**Affiliations:** Department of Surgery, Thomas Jefferson University Hospital, 1015 Walnut Street, 613 Curtis, Philadelphia, PA 19107 USA

**Keywords:** Percutaneous cholecystostomy tube, Interval cholecystectomy, Acute calculous cholecystitis

## Abstract

**Background:**

Percutaneous cholecystostomy (PCT) is an option for acute calculous cholecystitis in high-risk surgical patients. While PCT effectively manages acute episodes by providing source control, the management after PCT remains unclear. When feasible, subsequent interval cholecystectomy (IC) offers definitive disease resolution; however, clear guidelines for patient selection remain lacking. This study identifies factors that hinder the decision to proceed with IC, investigates whether IC after PCT is associated with improved survival, and assesses the incidence of subsequent biliary procedures after PCT.

**Methods:**

A retrospective cohort study was conducted using deidentified data from the TriNetX platform, encompassing over 100 million patients. Patients diagnosed with acute calculous cholecystitis who underwent PCT were identified. The primary outcome was the identification of factors associated with the failure to undergo IC after PCT. Secondary outcomes included assessing the hazard of death associated with IC, modeling IC as a time-varying covariate. Additionally, biliary interventions following PCT were quantified.

**Results:**

Among 419,102 patients with acute calculous cholecystitis, 8,483 (2.0%) underwent PCT. Of these, 43.0% subsequently underwent IC within one year. Patients with chronic ischemic heart disease, congestive heart failure, chronic obstructive pulmonary disease, ascites, diabetes, and concurrent diagnosis of septic shock were less likely to undergo IC. Additionally, 40.9% of patients required at least one additional biliary intervention within one year following PCT.

**Conclusion:**

More than half of patients do not undergo IC after PCT. Patients with comorbidities such as chronic ischemic heart disease, congestive heart failure, chronic obstructive pulmonary disease, ascites, diabetes, and concurrent diagnosis of septic shock are associated with failure to undergo IC. Moreover, patients who undergo PCT frequently require additional biliary interventions. This highlights the need for improved patient selection, structured follow-up, and optimization strategies to facilitate IC when feasible. A multidisciplinary approach is crucial for managing comorbidities, increasing surgical eligibility, and ultimately improving outcomes for patients undergoing PCT for acute calculous cholecystitis.

**Graphical abstract:**

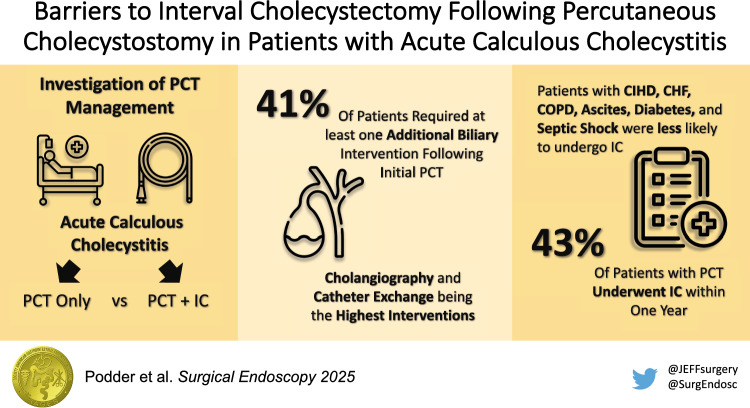

**Supplementary Information:**

The online version contains supplementary material available at 10.1007/s00464-025-12161-x.

Acute cholecystitis is a common gastrointestinal condition, affecting over 200,000 people annually in the United States (US) [1]. It is characterized by inflammation of the gallbladder, typically caused by cystic duct obstruction due to gallstones [1]. Cholecystectomy is the gold standard treatment, with laparoscopic cholecystectomy having largely replaced open cholecystectomy as the preferred approach [2]. Over 750,000 cholecystectomies are performed annually in the US [3, 4]. For patients with acute cholecystitis, percutaneous cholecystostomy (PCT) offers a safe, minimally invasive treatment approach for patients with exceptionally high perioperative risk [5, 6]. While PCT is widely accepted as a viable initial option for managing acute cholecystitis in patients unfit for surgery, the optimal algorithm following PCT remains uncertain in terms of patient follow up, patient selection and timing of the definitive procedure [7]. Patient selection for interval cholecystectomy (IC), cholecystectomy after PCT, is challenging and dynamic, as those treated with PCT likely had significant comorbidities and high surgical risk at the time of initial tube placement [8–11].

PCT lowers the initial perioperative risk of morbidity and mortality in select populations [12], however, its long-term effectiveness in alleviating symptoms of acute calculous cholecystitis remains debated [13, 14]. Studies indicate that failure to proceed with IC after PCT may lead to an increase in the risk of recurrent biliary interventions, hospital readmissions, morbidity and mortality [14, 15]. However, some patients may not be eligible for eventual IC due to factors such as advanced age, severe comorbidities, or persistent organ system dysfunction, the very reasons that led to PCT initially. Additionally, some patients may not achieve adequate optimization for surgery or may be lost to follow-up. Furthermore, the long-term burden of PCT, including the need for subsequent biliary interventions such as endoscopic or percutaneous procedures, is not well characterized.

The aim of this study was to determine (1) the clinical conditions that lead to PCT, (2) the clinical conditions associated with the failure to undergo IC, (3) whether IC after PCT is associated with improved survival, and (4) the incidence of biliary procedures following PCT in patients with acute calculous cholecystitis. Understanding these factors will guide clinical decision-making, improve outcomes for patients undergoing PCT for acute calculous cholecystitis, and promote streamlined follow-up and optimization for those who may benefit from eventual IC.

## Materials and methods

### Data source

This retrospective, population-based cohort study used deidentified data from TriNetX (TriNetX LLC, Cambridge, MA), a global health-collaborative clinical-research platform that collects real-time data from 105 healthcare organizations across the world. The TriNetX platform includes diverse patient data from over 100 million individuals. Data was collected from TriNetX on June 20, 2024. This study was evaluated by the Thomas Jefferson University Institutional Review Board and deemed exempted as it used only deidentified population-level records.

### Study population

This study utilized diagnostic and procedure codes from the International Statistical Classification of Diseases and Related Health Problems, Ninth Revision (ICD-9), Tenth Revision (ICD-10), and Common Procedural Terminology (CPT) to define our cohorts. All patients had a principal diagnosis of acute calculous cholecystitis. Supplemental Table 1 describes the codes used. Patients with the following criteria were excluded: 1) age < 18 years; 2) living outside of the United States; 3) having gallbladder, pancreatic, or hepatobiliary malignancy.

**Table 1:** Demographic and clinical characteristics of patients who were diagnosed with acute calculous cholecystitis (n = 419,102)

**Demographics**	
**Age, median (IQR)**	51 (36, 65)
**Gender**	
Male, n (%)	128,729 (32.2)
Female, n (%)	271,645 (67.8)
**Race**	
White, n (%)	280,498 (66.9)
African American, n (%)	37,650 (9.0)
Asian, n (%)	18,016 (4.3)
**Location**	
Midwest, n (%)	62,889 (15.0)
Northeast, n (%)	121,806 (29.1)
South, n (%)	143,418 (34.2)
West, n (%)	59,193 (14.1)
**Health History**	
Obesity, n (%)	130,805 (31.2)
Diabetes, n (%)	78,017 (18.6)
Hypertension, n (%)	165,489 (39.5)
Hyperlipidemia, n (%)	41,301 (9.9)
CIHD, n (%)	46,668 (11.1)
CHF, n (%)	28,980 (6.9)
COPD, n (%)	41,124 (9.8)
Ascites, n (%)	11,653 (2.8)
Smoking history, n (%)	13,088 (3.1)
Concurrent sepsis, n (%)	19,481 (4.6)
Concurrent septic shock, n (%)	5776 (1.4)
Chemotherapy within 30 days, n (%)	43,095 (10.3)
Radiotherapy within 90 days, n (%)	600 (0.1)
Percutaneous cholecystostomy, n (%)	8483 (2.0)

*CIHD*, Chronic ischemic heart disease; *CHF*,Congestive heart failure;*COPD*,Chronic obstructive pulmonary disease

**Fig. 1:**
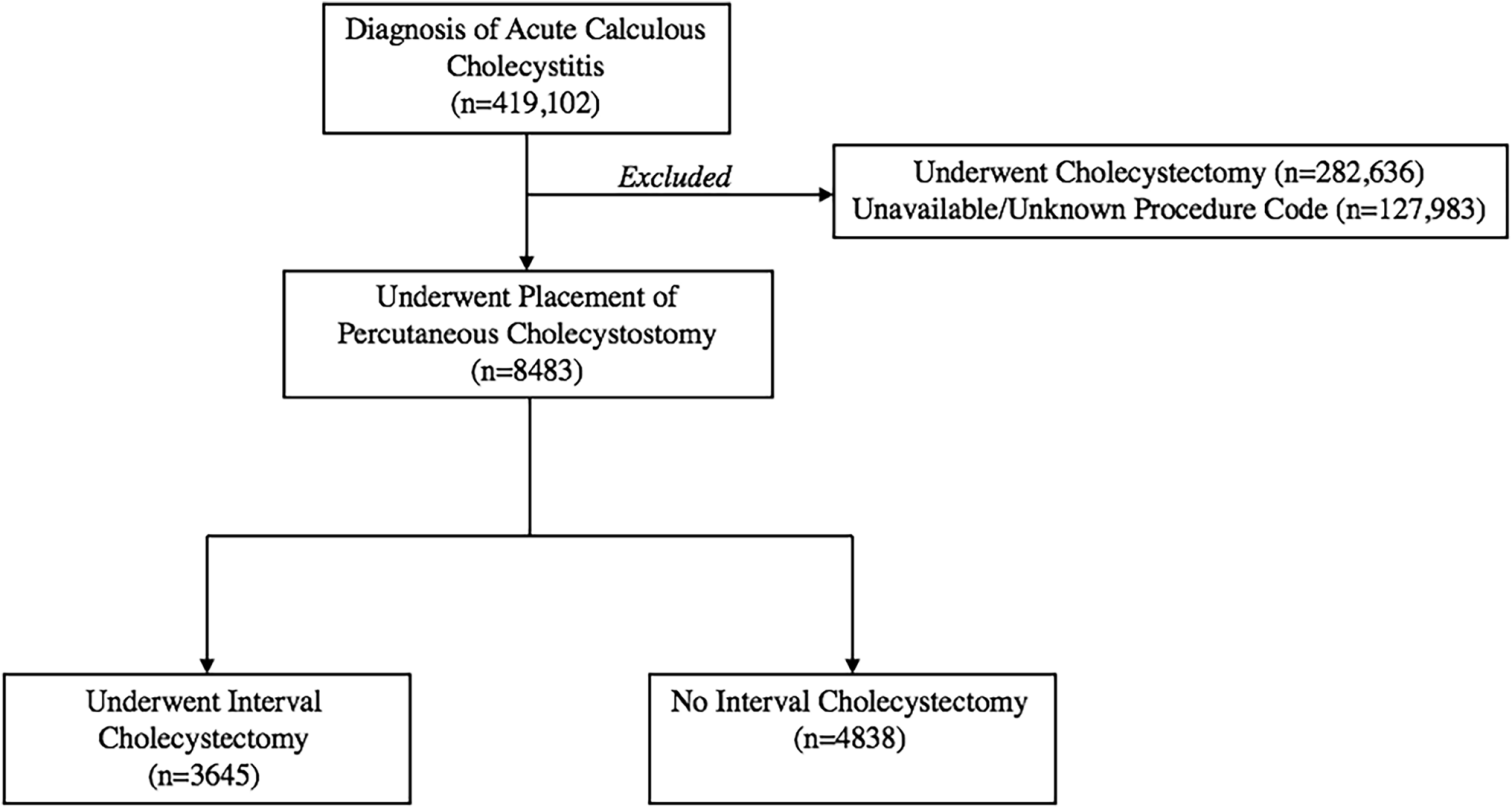
Representative diagram of management pathways for acute calculous cholecystitis in this analysis

The control cohort included patients with acute calculous cholecystitis and a primary procedure code for PCT placement within 30 days of cholecystitis diagnosis and no subsequent procedure code for cholecystectomy. The case cohort included patients with acute calculous cholecystitis with a primary procedure code for PCT within 30 days of cholecystitis diagnosis and subsequent procedure code for cholecystectomy (Supplemental Table 1). Figure 1 describes the management pathways for patients with acute calculous cholecystitis in this study.

### Outcomes and covariates

The primary outcome of this study was identifying factors associated with the failure to undergo IC after PCT. Secondary outcomes included identifying predictors for PCT placement, as well as mortality and survival following IC. The frequency of interventional radiology biliary procedures following PCT was analyzed, along with an attempt to evaluate the rate of IC and death after these procedures. A list of biliary procedure codes can be found in Supplemental Table 1.

Time to interval cholecystectomy was calculated from date of index PCT to the date of IC. Patient-level variables included age and sex. Comorbid conditions identified before the diagnosis of acute calculous cholecystitis included chronic ischemic heart disease (CIHD), congestive heart failure (CHF), chronic obstructive pulmonary disease (COPD), ascites, smoking history, type II diabetes, obesity, history of chemotherapy within 30 days, and history of radiation therapy within 90 days. Additionally, diagnoses of sepsis, severe sepsis, or septic shock occurring concurrently with or within 72 h of the acute calculous cholecystitis diagnosis were identified. Sepsis and severe sepsis were combined and captured as “concurrent diagnosis of sepsis”, while septic shock was captured as “concurrent diagnosis of septic shock”.

Due to the Trinetx platform being an administrative dataset, loss to follow-up cannot be directly determined. Instead, we defined the last available encounter or record in the dataset as the patient’s final point within the database.

### Statistical analysis

A Cox proportional hazards model was used to estimate the effect of interval cholecystectomy on mortality risk, incorporating the additional covariates listed above. IC was modeled as a time-varying covariate, assigned a value of 0 prior to surgery and 1 afterward, ensuring accurate classification of patients based on their surgical status at each time point. This was done to reduce immortal time bias. To identify predictors of PCT placement, a logistic regression model was used to assess the odds of undergoing PCT within 30 days of initial acute calculous cholecystitis diagnosis, incorporating patient-level variables and comorbidities. This analysis only included patients that could be followed for 30 days following initial acute calculous cholecystitis diagnosis. A separate logistic regression model evaluated factors influencing the likelihood of IC within 365 days after initial PCT placement. This analysis only included patients that could be followed for 365 days following initial PCT placement. Next, the mean and median number of biliary procedures following PCT placement were also calculated. Lastly, we quantified the outcomes of patients who underwent biliary intervention, categorizing their trajectory as death, interval cholecystectomy, or last available encounter in the dataset.

Given the exploratory nature of this analysis, P-values are not reported because a pre-specified adjustment for multiple comparisons was not applied [16]. For handling, missing data, a complete case analysis was utilized. This method utilizes only the cases with no missing values for covariates at baseline. As a result, patients were required to have fully observed data for both the outcome and all predictors in order to be included in each logistic regression model and Cox proportional hazards model. This method was chosen as predictor and outcome missingness were both scarce, and thus missingness was assumed to be negligible with regard to bias. All analyses were performed using R version 4.3.1 (R Foundation for Statistical Computing, Vienna, Austria).

## Results

### Patient demographics

There were 419,102 total patients that met our inclusion criteria and were diagnosed with acute calculous cholecystitis. The median age was 51 (IQR: 36–65) and the majority were female (67.8%) and white (66.9%). Comorbidities such as hypertension (39.5%), obesity (31.2%), and diabetes (18.6%) were common (Table 1).

Of these patients with acute calculous cholecystitis, 8,483 (2.0%) underwent PCT, while 282,636 (67.4%) underwent cholecystectomy and 127,983 (30.6%) did not have a procedure code for either cholecystectomy or PCT. Among those who received PCT, median age was 69 (IQR: 59–78), with a higher proportion of male patients (58.7%) and a higher burden of comorbidities, including CHF (37.0%), CIHD (41.7%), hypertension (71.8%), obesity (38.7%), diabetes (46.3%), and a concurrent diagnosis of sepsis (44.8%) (Table 2).

**Table 2:** Demographic and clinical characteristics of patients who underwent percutaneous cholecystostomy (n = 8483)

**Demographics**	
**Age, median (IQR)**	69 (59, 78)
**Gender**	
Male, n (%)	4804 (58.7)
Female, n (%)	3374 (41.3)
**Race**	
White, n (%)	5645 (66.5)
African American, n (%)	949 (11.2)
Asian, n (%)	402 (4.7)
**Location**	
Midwest, n (%)	1475 (17.4)
Northeast, n (%)	2568 (30.3)
South, n (%)	2901 (34.2)
West, n (%)	1279 (15.1)
**Health History**	
Obesity, n (%)	3280 (38.7)
Diabetes, n (%)	3929 (46.3)
Hypertension, n (%)	6092 (71.8)
Hyperlipidemia, n (%)	1771 (20.9)
CIHD, n (%)	3539 (41.7)
CHF, n (%)	3142 (37.0)
COPD, n (%)	2117 (25.0)
Ascites, n (%)	1081 (12.7)
Smoking history, n (%)	508 (6.0)
Concurrent sepsis, n (%)	3802 (44.8)
Concurrent septic shock, n (%)	1766 (20.8)
Chemotherapy within 30 days, n (%)	2011 (23.7)
Radiotherapy within 90 days, n (%)	82 (1.0)
Interval cholecystectomy, n (%)	3645 (43.0)
Days to interval cholecystectomy, median (IQR)	54 (25, 93)
Death within one year without interval cholecystectomy, n (%)	2110 (24.9)
Days to death, median	44

*CIHD*,Chronic ischemic heart disease; *CHF*, Congestive heart failure; *COPD*,Chronic obstructive pulmonary disease

Among patients who underwent PCT, 43.0% (n = 3,645) subsequently underwent IC within one year, with a median time from PCT to IC of 54 days (IQR: 25–93) (Table 2). Additionally, 24.9% (n = 2110) of patients died, without having undergone IC, with a median time from PCT to death of 44 days. The remaining 32.1% (n = 2,728) had no further records available in the database, limiting our ability to assess their outcomes.

### Predictors for percutaneous cholecystostomy

**Fig. 2:**
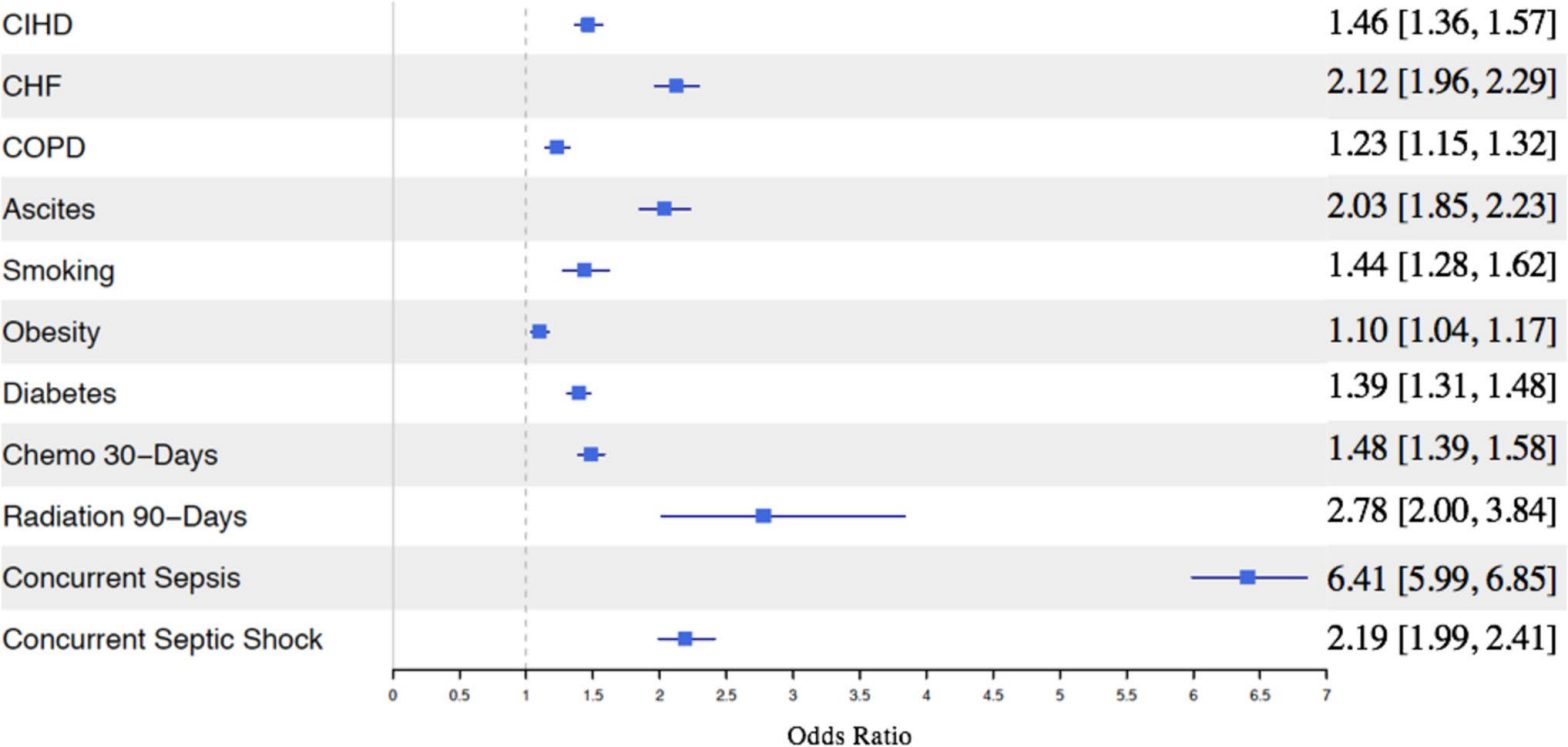
Health covariates associated with receiving a percutaneous cholecystostomy

Patients with all comorbidities studied were associated with higher odds of receiving a PCT within 30 days after a diagnosis of acute calculous cholecystitis. The logistic regression model estimated that patients with a concurrent diagnosis of sepsis (OR: 6.41, 95% CI: [5.99, 6.85]), history of radiation therapy within 90 days (OR: 2.78, 95% CI: [2.00, 3.84]), concurrent diagnosis of septic shock (OR: 2.19, 95% CI: [1.99, 2.41]), CHF (2.12, 95% CI: [1.96, 2.29]) and ascites (2.03, 95% CI: [1.85, 2.23]) had the highest odds of receiving PCT (Fig. 2).

### Predictors for interval cholecystectomy

**Fig. 3:**
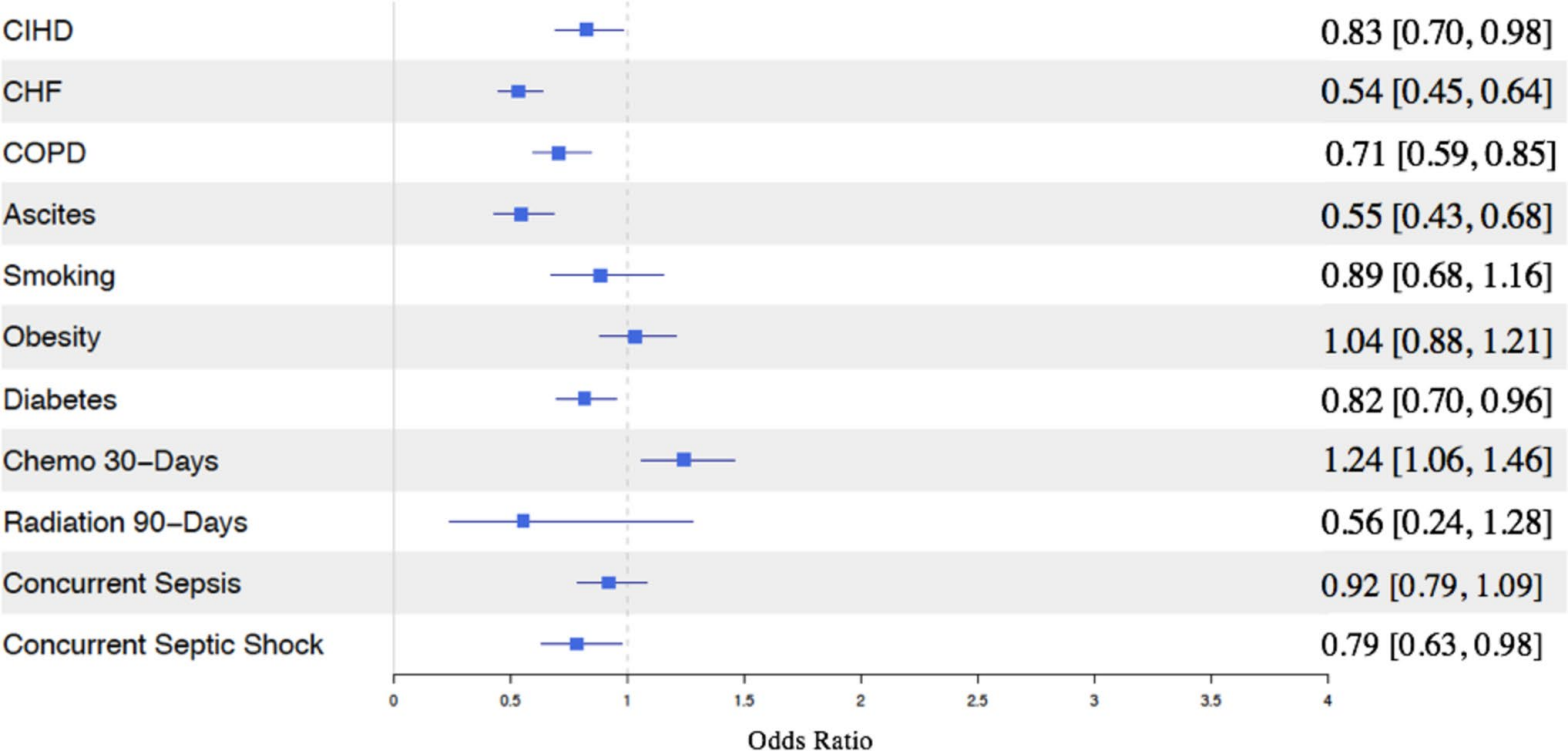
Health covariates associated with undergoing an interval cholecystectomy

The logistic regression modeling analysis identified predictors for undergoing IC within one year of PCT. Chemotherapy within 30 days (OR: 1.24, 95% CI: [1.06, 1.46]) was associated with a higher likelihood of undergoing IC. Conversely, CIHD (0.83, [0.70, 0.98]), CHF (0.54, [0.45, 0.64]), COPD (0.71, [0.59, 0.85]), ascites (0.55, [0.43, 0.68]), diabetes (0.82, [0.70, 0.96]), and concurrent diagnosis of septic shock (0.79, [0.63, 0.98]) were associated with lower odds of IC (Fig. 3).

### Survival analysis

**Fig. 4:**
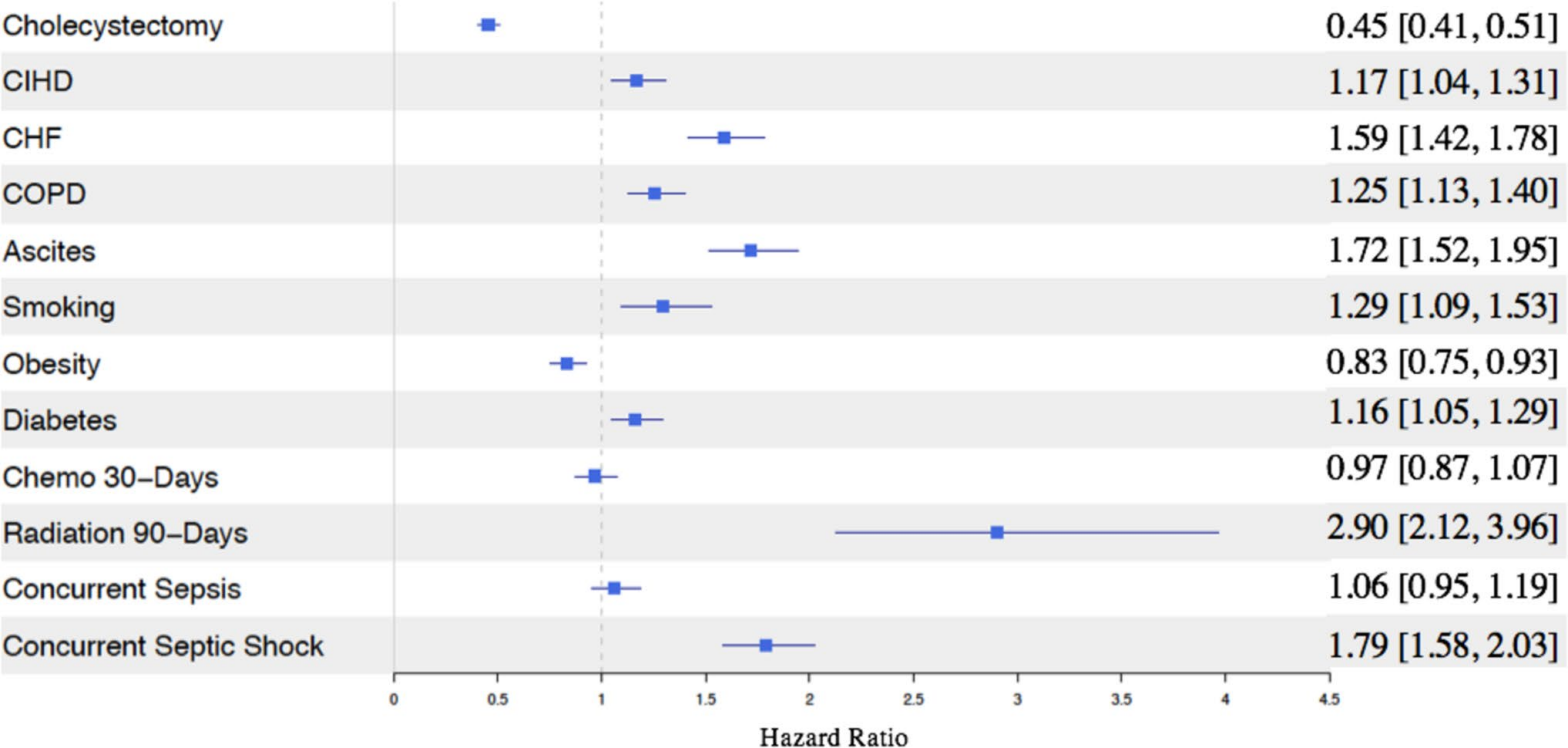
Impact of interval cholecystectomy and health covariates on survival after percutaneous cholecystostomy

Cox proportional hazards modeling analysis demonstrated that IC was associated with a significant reduction in mortality (HR: 0.45, 95% CI: 0.41–0.51). Conversely, concurrent diagnosis of septic shock at the time of PCT was associated with a significant increase in mortality (1.79, [1.58, 2.03]) (Fig. 4).

### Biliary procedures following percutaneous cholecystostomy

Slightly under half of patients (40.9%, n = 3471) who underwent PCT required at least one additional biliary procedure within one year, with a median of 0 (IQR: 0–1) procedures and a mean of 0.86. The most common interventions included cholangiography (CPT 47531) and biliary drainage catheter exchange (CPT 47536). Among patients who required at least one biliary intervention post-PCT, the median number of procedures was 1 (IQR: 1–2) and a mean of 2.09 (Table 3).

**Table 3:** Procedure counts and descriptive summary for biliary procedures after percutaneous cholecystostomy

Procedure Codes	Procedure Count (%)
47531, Injection procedure for cholangiography through an existing access or catheter	3060 (42.2%)
47532, Injection procedure for cholangiography through an existing access or catheter	71 (1.0%)
47533, Placement of biliary drainage catheter, percutaneous, including diagnostic cholangiography when performed, imaging guidance	36 (0.5%)
47534, Placement of biliary drainage catheter, percutaneous, including diagnostic cholangiography when performed, imaging guidance	44 (0.6%)
47535, Conversion of external biliary drainage catheter to internal‐external biliary drainage catheter	33 (0.5%)
47536, Exchange of biliary drainage catheter	3389 (46.7%)
47537, Removal of biliary drainage catheter, percutaneous, requiring fluoroscopic guidance	440 (6.1%)
47538, Placement of stent(s) into a bile duct	11 (0.2%)
47542, Balloon dilation of biliary ducts(s) or of ampulla	44 (0.6%)
47544, Removal of calculi/debris from biliary duct(s) and/or gallbladder	128 (1.8%)

Summary Statistics	Mean	Median (IQR)
Biliary procedures counts after PCT	0.86	0 (0,1)
Biliary procedures counts after PCT for patients with ≥ 1 procedure	2.09	1 (1,2)

Additionally, among patients who underwent at least one biliary procedure following PCT, 44.3% (n = 1,536) subsequently underwent IC, with a median time to IC of 76 days after PCT. In contrast, 22.4% (n = 778) died before receiving IC, with a median time to death of 179 days post-PCT. The remaining 33.3% (n = 1,157) neither died nor underwent IC and were lost in the database at a median of 365 days after PCT (Table 4).

**Table 4:** IC and death rate within 1 year of PCT in patients with biliary procedures (n = 3471)

Outcome	Count (%)	Median Days to Outcome
Interval Cholecystectomy	1536 (44.3%)	76
Death	778 (22.4%)	179
Lost from Database*	1157 (33.3%)	365

^*^These patients were no longer captured in the database and could not be followed

## Discussion

In this analysis, we show that more than half of patients do not undergo interval cholecystectomy after percutaneous cholecystostomy. Patients with comorbidities such as chronic ischemic heart disease, congestive heart failure, chronic obstructive pulmonary disease, ascites, diabetes, and concurrent diagnosis of septic shock are associated with a failure to undergo IC. Furthermore, a significant proportion require ongoing biliary interventions, underscoring the need for careful patient selection, clear guidelines for subsequent IC, multidisciplinary optimization, and structured follow-up strategies to ensure timely and appropriate surgical management after PCT.

While PCT placement is a treatment option for acute cholecystitis in critically ill patients or patients with a high surgical risk, consensus on post-PCT management is lacking. When feasible, subsequent IC provides complete disease resolution particularly in patients with gallstones. However, existing literature has yet to provide clear guidelines on patient selection for eventual IC or the best approach to managing patients following PCT placement [15]. Addressing these gaps is crucial in optimizing outcomes and minimizing complications in this high-risk population.

This analysis of 419,102 patients with acute calculous cholecystitis in the TriNetX database found that 2% (n = 8,483) underwent percutaneous cholecystostomy (PCT), consistent with prior studies using the National Inpatient Sample (2005–2014), which reported a rate of 2.9% [17]. Among PCT recipients, the median age was 69 years, they were predominantly male patients (58.7%), they had a higher burden of comorbidities, and they presented critically ill, with 20.8% presenting with septic shock. These findings reinforce existing literature, indicating that patients undergoing PCT tend to be older, predominantly male, have a greater burden of comorbidities, and are more acutely ill on presentation.

This analysis also demonstrates that only 43.0% of patients who underwent PCT subsequently underwent IC within one year, which is in line with prior literature reports demonstrating rates ranging from 28 to 55% (Table 2). [8, 10, 15, 18–21]. Several clinical factors contributed to the failure to undergo definitive surgery. Our analysis found that patients with CIHD, CHF, COPD, ascites, diabetes, and concurrent diagnosis of septic shock had lower odds of undergoing IC (Fig. 3). Additionally, one in four patients undergoing PCT, without eventual IC, died within one year, with a median time to death of 44 days. This again reflects the older age, higher burden of comorbidities, and greater acuity among patients undergoing PCT, many of whom may never recover enough to undergo definitive surgical management.

Interestingly, patients who had undergone chemotherapy within 30 days had higher odds of undergoing IC. We believe this may be because recent chemotherapy does not necessarily preclude a patient from undergoing surgery, whereas the other medical factors associated with lower odds (i.e. CIHD, CHF, COPD) may be stronger indicators of frailty or critical illness, making the patient less likely to be eligible for surgery. Additionally, prior literature has identified Tokyo Guidelines 2018 (TG18) Grade III severity for acute cholecystitis and hypoproteinemia as factors negatively influencing the decision to pursue IC [22]. Interestingly, they identify a history of malignancy as a negative predictor for undergoing IC, which contradicts our findings. However, this discrepancy may be due to the distinction in definition between patients with a history of malignancy and those actively undergoing chemotherapy within 30 days, as these represent different patient populations.

The lack of definitive treatment in these patients likely reflects the same clinical concerns that initially led to PCT rather than upfront cholecystectomy [23]. These findings highlight the importance of a multidisciplinary approach to patient selection and optimization for eventual IC. Collaboration amongst surgeons, anesthesiologists, and other medical specialists is essential to balancing operative risk with the potential benefits of definitive treatment. Beyond surgical considerations, individualized management strategies should be tailored to patient-specific factors, with close follow-up to ensure ongoing care and oversight. Maximizing clinical expertise through guidelines and optimizing institutional resources can further enhance patient outcomes [24]. Lastly, patients with such complex clinical scenarios may benefit from management at institutions with experience in caring for this population.

This analysis also demonstrates that IC following PCT is associated with a significant reduction in mortality compared to patients undergoing PCT alone, even after adjusting for multiple comorbidities. In our cohort, patients who underwent IC had a hazard ratio for mortality of 0.45 (95% CI: 0.41–0.51) as compared to identical patients who did not undergo IC (Fig. 4). Similarly, a single-institution study also reported significantly improved survival among patients who underwent IC compared to those managed with PCT alone [15]. These findings suggest that, whenever feasible, IC should be prioritized as the standard of care following PCT especially in patients with acute calculous cholecystitis. Though, the survival benefit with IC may, in part, reflect selection bias, as patients undergoing IC likely have fewer severe comorbidities than those managed with PCT alone. Thirty-day mortality rates range from 9 to 39% [15, 23], largely reflecting the severity of illness in patients undergoing PCT, which prevents them from undergoing IC.

The low rate of subsequent IC may also reflect the hesitancy towards surgery due to the difficulty of surgery after PCT [23]. With that said, surgeons managing patients with PCT, who eventually offer cholecystectomy, should be aware of the increased risk of an open procedure [25, 26]. They should have detailed preoperative discussions with patients to set appropriate expectations [15]. Additionally, intraoperative strategies such as subtotal cholecystectomy may be used in cases where unclear anatomy may make complete gallbladder removal unsafe. This highlights the need for clearer recommendations on surgical decision-making after PCT and appropriate treatment at institutions with expertise in managing these high-risk patients.

Another finding in this study is that 40.9% of patients who underwent PCT required at least one additional biliary procedure within one year. The most common interventions were cholangiography (42.2%) and biliary drainage catheter exchange (46.7%). Although the median number of biliary procedures was 1 among patients with at least one biliary procedure, the upper quartile range extends to 2, indicating that a subset of patients undergoes multiple biliary procedures post-PCT (Table 3). These findings suggest that while PCT offers immediate relief of obstruction, it often leads to recurrent biliary complications and repeated interventions, which places a burden on both patients and healthcare resources. This reinforces the importance of transitioning to IC when clinically feasible, as definitive surgical management has been shown to reduce the long-term need for additional interventions. Alvino et al. demonstrated that IC reduced the risk of recurrent biliary events to 7% from 21% [14].

Furthermore, patients with PCT remain at increased risk for morbidity and mortality. Even among those receiving at least one biliary procedure after PCT, fewer than half (44.3%) proceed to IC, and a substantial proportion either die (22.4%) or exit the database without clear outcomes (Table 4). Future studies should aim to refine criteria for IC candidacy, help identify patients who can benefit from a multidisciplinary approach for mitigation of comorbid conditions to improve patient outcomes while minimizing healthcare costs.

Unfortunately, not all patients will be candidates for IC, even after thorough medical optimization and multidisciplinary evaluation. For this subset of high-risk patients, clinical decisions must be individualized centering around symptom control and careful follow-up. A trial of PCT removal may be appropriate in select cases, however, some patients may experience recurrence of cholecystitis. Where feasible, referral to a tertiary or quaternary care center may be appropriate, especially for institutions lacking the resources to manage complex biliary disease in medically fragile patients. And in a minority of cases, advanced endoscopic interventions such as endoscopic transpapillary gallbladder drainage (ETGBD) or endoscopic ultrasound-guided gallbladder drainage (e.g., EDGE procedures) may serve as alternatives to cholecystectomy [27, 28]. Ultimately, the management of patients with no clear path to IC must balance the risks of intervention against the patient’s overall prognosis, quality of life, and goals of care.

Given the biliary complications, readmissions, and higher rates of open cholecystectomy, it raises the question of whether PCT is being over utilized. The randomized trial (CHOCOLATE Trial), which compared high-risk patients with acute calculous cholecystitis undergoing either laparoscopic cholecystectomy or PCT, found that laparoscopic cholecystectomy significantly reduced the rate of major complications compared to percutaneous catheter drainage [29]. Future research should focus on refining patient selection criteria for PCT to ensure it is appropriately utilized.

This study is not without limitations. First, given this is a retrospective study it is subject to selection bias. Second, the available data may not fully capture the complete clinical picture that influenced specific management decisions, such as the choice to proceed with IC or pursue additional biliary interventions. Factors such as surgeon preference, institutional protocols, and nuanced patient-specific considerations including prior abdominal surgeries or physiologic derangements may not be adequately accounted for in our analysis. Additionally, the dataset lacks hospital identifiers, referral patterns, and transfer information, limiting our ability to assess care settings or structural differences in care delivery. Third, give the limitations of the database, we are unable to accurately comment on more nuanced outcomes such as rate of readmissions, common bile duct injury during IC, conversion rates from laparoscopic to open, and other surgery specific outcomes. Lastly, we are unable to determine the definitive treatment outcomes for patients no longer captured in the database after a certain period. While we assessed the duration of their presence in the dataset, we cannot comment on their subsequent clinical course.

Despite these limitations, this study possesses several strengths. Most noticeably, this study includes data from multiple healthcare organizations from the TriNetX platform which contains patient data from over 100 million individuals. This allows for a representative population of patients to be included and minimizes possible institutional biases, which provides data to generalize the observations to clinical practice in the US. Additionally, the large sample size provided by this database enables robust statistical analyses, improves the reliability of findings, and allows for a more comprehensive evaluation of factors influencing patient outcomes.

## Conclusions

This study demonstrates that more than half of patients do not undergo IC due to advanced comorbidities, and a substantial proportion require ongoing biliary interventions after PCT. These findings highlight the importance of careful patient selection for PCT, implementation of structured follow-up strategies, and multidisciplinary optimization to facilitate IC for patients who have undergone PCT. Ensuring appropriate patient optimization to enable definitive surgical management may be important for reducing healthcare utilization and improving the quality of life for this high-risk patient population.

## Supplementary Information

Below is the link to the electronic supplementary material.

Supplementary file1 (DOCX 65 KB)
