# Correction: Barriers to interval cholecystectomy following percutaneous cholecystostomy in patients with acute calculous cholecystitis

**DOI:** 10.1007/s00464-025-12296-x

**Published:** 2025-10-10

**Authors:** Sourav Podder, Kirsten Lung, George Ibrahim, Scott Koeneman, Joshua Marks, Murray Cohen, Anirudh Kohli

**Affiliations:** Department of Surgery, Thomas Jefferson University Hospital, 1015 Walnut Street, 613 Curtis, Philadelphia, PA 19107 USA

**Correction to: Surgical Endoscopy** 10.1007/s00464-025-12161-x

The original online version of this article was revised to correct the artwork used for Fig. 1.

**Fig. 1:**
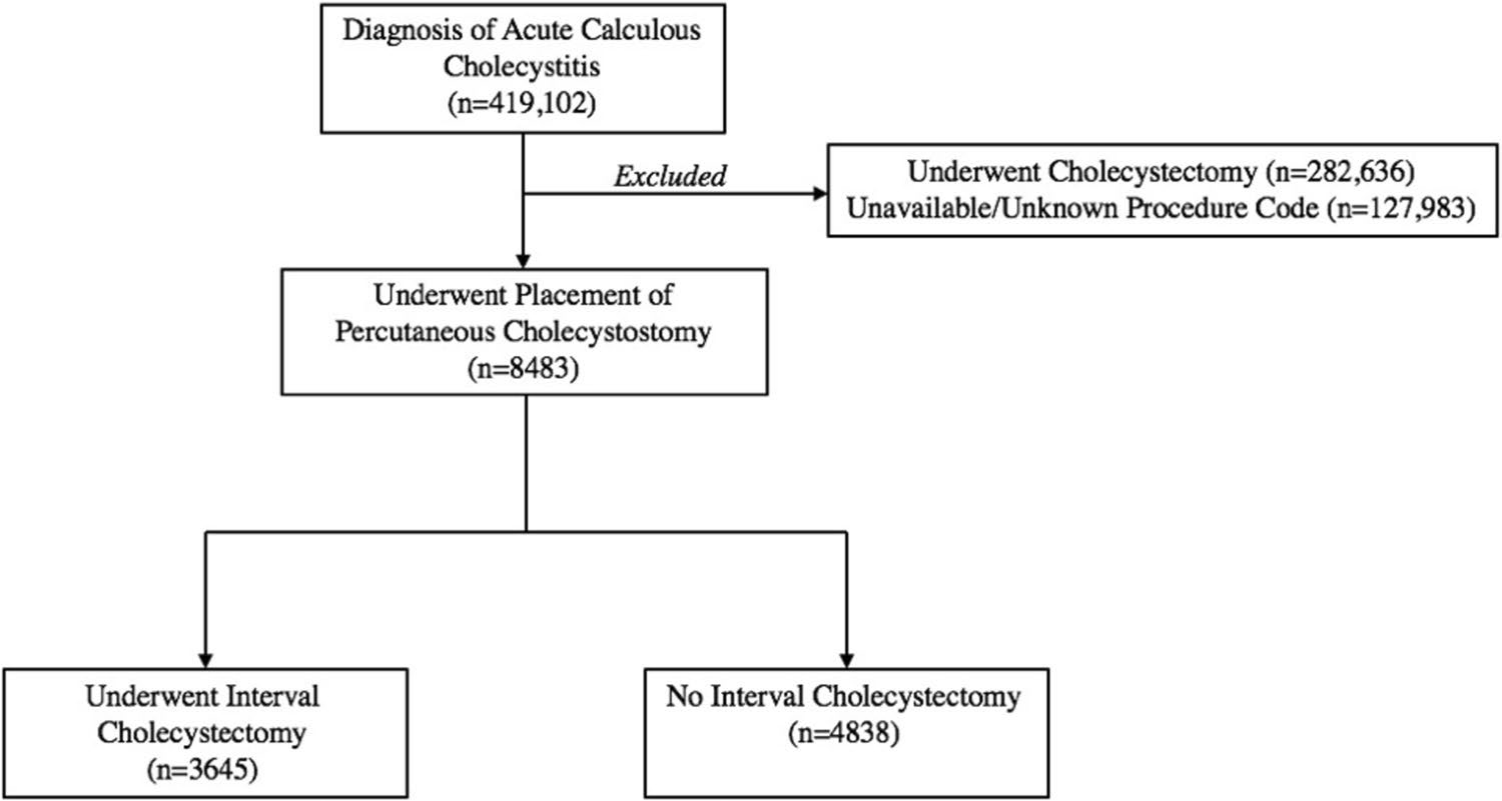
Representative diagram of management pathways for acute calculous cholecystitis in this analysis

