# Supplementary material for: Barriers to interval cholecystectomy following percutaneous cholecystostomy in patients with acute calculous cholecystitis
Source: Surg Endosc. 2025 Sep 22;39(12):8488–97. doi: 10.1007/s00464-025-12161-x (PMC12708813; doi:10.1007/s00464-025-12161-x)
Supplement: Supplementary file 1 — Supplementary file1 (DOCX 65 KB) [file 464_2025_12161_MOESM1_ESM.docx]

Table S1a. Codes used for diagnosis of Acute Calculous Cholecystitis

| **Acute Calculous Cholecystitis** | |
| --- | --- |
| ICD-9-CM | ICD-10-CM |
| 574.0 - Calculus of gallbladder with acute cholecystitis | K80.0 – Calculus of gallbladder with acute cholecystitis |
| 574.1 - Calculus of gallbladder with other cholecystitis | K80.1 – Calculus of gallbladder with other cholecystitis |
| 574.3 - Calculus of bile duct with acute cholecystitis | K80.4 – Calculus of bile duct with cholecystitis |
| 574.6 - Calculus of gallbladder and bile duct with acute cholecystitis | K80.60 – Calculus of gallbladder and bile duct with cholecystitis |
| 574.4 - Calculus of bile duct with other cholecystitis |  |
| 574.7 - Calculus of gallbladder with other cholecystitis |  |
| 574.8 - Calculus of gallbladder and bile duct with acute and chronic cholecystitis |  |

Table S1b. Codes used to determine Cholecystectomy and Percutaneous Cholecystostomy

| Procedure | ICD-9-CM | ICD-10-PCS | CPT |
| --- | --- | --- | --- |
| **Open Cholecystectomy** |  |  |  |
| Open cholecystectomy | 51.2, 51.22 | 0FB40ZZ, 0FT40ZZ | 47600 |
| Cholecystectomy with cholangiography (Open) |  |  | 47605 |
| Cholecystectomy with exploration of common duct (Open) |  |  | 47610 |
| Cholecystectomy with choledochoenterotomy (Open) |  |  | 47612 |
|  | | | |
| **Laparoscopic Cholecystectomy** |  |  |  |
| Laparoscopic cholecystectomy | 51.23 | 0FB43ZZ, 0FB44ZZ, 0FT44ZZ | 47562 |
| Cholecystectomy with cholangiography | 51.21 |  | 47563 |
| Cholecystectomy with exploration of common duct | 51.24 |  | 47564 |
|  | | | |
| **Percutaneous Cholecystostomy** |  |  |  |
| Percutaneous Cholecystostomy | 51.0 | 0F9430Z | 47490 |

Table S1c. Codes used to determine interventional radiology biliary interventions

| **CPT** | **Biliary Interventions** |
| --- | --- |
| 47531 | Injection procedure for cholangiography through an existing access or catheter |
| 47532 | Injection procedure for cholangiography through an existing access or catheter |
| 47533 | Placement of biliary drainage catheter, percutaneous, including diagnostic cholangiography when performed, imaging guidance |
| 47534 | Placement of biliary drainage catheter, percutaneous, including diagnostic cholangiography when performed, imaging guidance |
| 47535 | Conversion of external biliary drainage catheter to internal‐external biliary drainage catheter |
| 47536 | Exchange of biliary drainage catheter |
| 47537 | Removal of biliary drainage catheter, percutaneous, requiring fluoroscopic guidance |
| 47538 | Placement of stent(s) into a bile duct |
| 47542 | Balloon dilation of biliary ducts(s) or of ampulla |
| 47544 | Removal of calculi/debris from biliary duct(s) and/or gallbladder |
